# Honors to the Dead

**Published:** 1863-03

**Authors:** 


### HONORS TO THE DEAD.

A meeting of the commissioned officers of the 9th regiment Illinois cavalry, was held at the headquarters of the regiment, Helena, Arkansas, on the 21st of February. A preamble and resolutions, testifying their sincere respect for the late Surgeon Charles Brackett, who died at that place on the 21st, were unanimously passed as follows:

*Resolved*, That in his death, we have lost not only an accomplished and scientific surgeon, who labored among us with the most self-sacrificing zeal, but a friend endered to us all by his pure and blameless life, and that we will cherish the memory of his good deeds, his great heart, and his earnest truthfulness.

*Rssolved*, That in his dying words, "I am glad that I have always tried to do right," a rich legacy to his children, we recognize the true character of the man, who from the most exalted patriotism, risked his feeble remnant of life in the service of his country, and died a martyr to his profession and his country's cause.

Resolutions to wear the usual badge of mourning, &c., were adopted.

We take the above notice of the death of Dr. Brackett from the Chicago *Tribune* of the 18th April. The doctor was for many years an occasional contributor to the pages of this Journal, and will be remembered as a writer of unusual originality and clerness. His devotion to the profession was only exceeded by that to his country, whose service he entered at an early period of the war.

Among the thousands of martyrs to the good cause not one was more deserving than he, and the members of the profession will join with the officers of the 9th Illinois Cavalry in marks of regret at his loss and in tenders of sympathy to his friends and family. D. B.

---

ANN ARBOR, Mich., March 1st, 1863.

MESSRS. EDITORS:

It is said *tempora mutantur et nos mutanur cum illis.* Do not believe it. The times change, but we continue unchanged and totally unmindful of the times. The nation is shattered to fragments—but what matter? We remain blissfully unconscious.

That which was only a rumor last month has since hardened into an event. I have heard that an announcement has been issued by the Chairman of the Com. of Arrangements, of an attempt to organize the *American* Medical Association on a sectional basis. The uneasy gentlemen who attempted this same action in 1861 and 1862, have succeeded thus far in 1863. They are literally seeking to expand their bubble reputation at the cannon's mouth. Hopeless of achieving more than a contemptible mediocrity in their respective home spheres, they strive to gain an arena where points of order and noise, intrigue and "deportment" may yield a factitious notoriety—easily to be mistaken by the groundlings for fame. We may expect floods of platitudes, and oceans of muddy wisdom. As the patenting of professinal tools of all sorts is prohibited by the code, we may expect to see sundry dray loads of splints, cutlery, stretching apparatus, and diagrams, on the floor, and in the ante-rooms of the Association, so that they may be carried from thence in triumph with its *Imprimatur* upon them.

The ambitious are already pricking up their ears as they read over the names of the offices to be filled. Our *quondam* friend P. is already arranging his glossy curls, and practicing before the mirror the attitudes he will strike and the gestures he will indulge—wondering, meanwhile, whether a Northern audience will be as much astounded to think that one small head could hold all he knew, as of yore, was that which now, alas, has gone away into Dixie's melancholy abyss of secession. Will he make the same speech here in the shadow of the Chicago Platform that he did there? And, if he does, will the Chairman of the Com. take up on that side or the other? But the subject grows upon us, and we forbear, with a few suggestions:

*First*, That the Com. reconsider their programme and fix the meeting at the same time with the great Canal Convention. This would be likely to ensure a better attendance.

*Second*, That as these are "ticklish times" and there are many angry men on both sides, each of the delegates be requested to come fully armed. This second suggestion is important, because everybody knows that the inevitable negro, and the war, will be lugged into the first, and probably every, speech made during the session.

*Third*, That the members who wish to advertise themselves, or their wares, be assessed to pay expenses. If they will travel, let them be voted Pickwickian consent by the Com.

*Fourth*, That the late President of the Ohio State Medical Society be requested to read his address on retiring from that position, to the Association and as many invited guests as the hall selected will contain. (The only objection to this suggestion is, that the second one above stated might thereby be more imperatively demanded, as a rule of order to the exclusion of the police.)

*Fifth*, That inasmuch as the exodus of so large a number of able-bodied men from Eastern and Middle States, which have not supplied their military quotas, to Illinois, which has surpassed its allotment, might be mistaken by the authorities as an attempt to escape the draft,—therefore let no delegates be admitted who have not passed the age of forty-five.

SOJOURNER.

[We beg leave to call the attention of our correspondent to the fact that the meeting is set down for the same day with that of the Grand Canal Convention. We suggest to him to bring along a tent and rations. We will divide our own shelter and food with visitors so far as they will go; but there is a physical limit which even our most expansive benevolence cannot pass.—ED.]

---

*Homœopathy.*—A recent graduate from Hahneman College in this city, a resident, we believe, of Sangamon county in this State, immediately after his graduation secured the position of contract surgeon at the Mound City Hospital.

The question arises how came this about? But for him there is no further question. It seems the poor boy really believed the twaddle he had been taught in the College. Soon after his entering upon his duties, he was attacked with "Congestive Chills" (Pernicious Remittent). The second day he died. There was nothing between that death and a long life but a few grains of Quinine. Pained as we always are at the death of young men, we can not after all but think of those soldiers whose lives will be saved from similar attacks ———!

But again we ask who is responsible for this gross outrage upon the profession—and what is beyond and above that, the rights and safety of our brave soldiery?

---

*The Clinics.*—Daily Clinics are attended at Rush Medical College, at 3 o'clock P. M. A large number of patients offer themselves for treatment, and the opportunity thus afforded to students is very superior. Our city is increasing so rapidly in population that this department of medical and surgical teaching is rapidly assuming great prominence. The facilities for practical illustration of the nature and treatment of both medical and surgical diseases, the ensuing session of the College, will be increased to a degree heretofore unattained at the West.

*A Glimpse of Army Practice.*—Elmer Nichols, M. D., of the 118th Ill. Vol., was detailed on the 24th of January to take charge of 328 sick on board the steamer J. C. Swan, *en route* from Vicksburg to St. Louis. From Vicksburg to Memphis they had neither medicines nor hospital stores in noticeable amount, but at Memphis a few supplies were secured. No assistant whatever was allowed him during the whole seven days' trip, notwithstanding he himself was scarcely recovered from recent sickness. So anxious were the regimental surgeons to get rid of their sick, that men were sent on board in an actually dying state. By working sixteen hours *per diem*, he could manage to give each patient an average of *two minutes and some seconds* attendance. *Seventy-six* died on the trip up!

This is the kind of labor imposed on those surgeons who are unhappy enough to wear Major's shoulder straps, and yet the abuse lavished upon them as a class would lead one to think their lives a perpetual holiday, and that they were rioting in luxury and ease.

---

*A New Excretory Function of the Liver.*—Prof. A. Flint, Jr., in the *Am. Jour.*, Oct. 1862, discusses the great importance of *Cholesterine* an as excrementitious substance, urging that: "What the discovery of the function of urea has done for diseases which now come under the head of uremia, the discovery of the function of Cholesterine may do for the obscure diseases which may hereafter be classed under the head of Cholesteremia."

In accordance with the opinion of the best physiologists, Prof. F. concludes the bile to be partly excrementitious and partly recrementitious. A series of carefully considered and managed experiments are given, and from their results, taken in connection with those made by other experimenters, he arrives at the following conclusions:

1. Cholesterine exists in the bile, the blood, the nervous matter, the crystalline lens, and the meconium, but does not exist in the feces in ordinary conditions. The quantity of cholesterine in the blood of the arm is from five to ten times more than the ordinary estimate.

2. Cholesterine is formed, in great part if not entirely, in the substance of the nervous matter, where it exists in great abundance, from which it is taken up by the blood, and constitutes one of the most important of the effete or excrementitious products of the body. Its formation is constant, it always existing in the nervous matter and the circulating fluid.

3. Cholesterine is separated from the blood by the liver, appears as a constant element of the bile, and is discharged into the alimentary canal. The history of this substance, in the circulating fluid and in the bile, marks it as a product destined to be gotten rid of by the system, or an excretion. It pre-exists in the blood, subserves no useful purpose in the economy, is separated by the liver and not manufactured there, and, if this separation be interfered with, accumulates in the system, producing blood-poisoning.

4. The bile has two separate and distinct functions dependent on the presence of two elements of an entirely different character. It has a function connected with nutrition. This is dependent on the presence of the glyco-cholate and tauro-cholate of soda, which do not pre-exist in the blood, subserve a useful purpose in the economy, and are not discharged from it, are manufactured in the liver and peculiar to the bile, do not accumulate in the blood when the function of the liver is interfered with, and are, in short, products of *secretion*. But it has another function connected with depuration, which is dependent on the presence of the cholesterine, which is an *excretion*. The flow of the bile is remittent, being much increased during the digestive act, but produced during the intervals of digestion, for the purpose of separating the cholesterine from the blood which is constantly receiving it.

5. The ordinary normal feces do not contain cholesterine, but contain *stercorine* (formerly called seroline, from its being supposed to exist only in the serum of the blood), produced by a transformation of the cholesterine of the bile during the digestive act.

6. The change of cholesterine into stercorine does not take place when digestion is arrested, or before this process commences; consequently, stercorine is not found in the meconium, or in the feces of hibernating animals during their torpid condition. These matters contain cholesterine in large abundance, which also sometimes appears in the feces of animals after a prolonged fast. Stercorine is the form in which cholesterine is discharged from the body.

7. The difference between the two varieties of jaundice with which we are familiar, the one characterized only by yellowness of the skin, and comparatively innocuous, while the other is attended with very grave symptoms, and is almost invariably fatal, is dependent upon the obstruction of the bile in the one case, and its suppression in the other. In the first instance, the bile is confined in the excretory passages, and its coloring matter is absorbed, while in the other, the cholesterine is retained in the blood, and acts as a poison.

8. There is a condition of the blood dependent upon the accumulation of cholesterine which I have called *Cholesteremia.* This only occurs when there is structural change in the liver, which incapacitates it from performing its excretory functions. It is characterized by symptoms of a grave character, referable to the brain, and dependent upon the poisonous effects of the retained cholesterine on this organ. It occurs with or without jaundice.

9. Cholesteremia does not occur in every instance of structural disease of the liver. Enough of the liver must be destroyed to prevent the due elimination of the cholesterine. In cases in which the organ is but moderately affected, the sound portion is capable of performing the eliminative function of the whole.

10. In cases of simple jaundice, when the feces are decolorized and the bile is entirely shut off from the intestine, stercorine is not found in the evacuations; but in cases of jaundice with cholesteremia, the stercorine may be found, though always very much diminished in quantity, showing that there is an insufficiency in the separation of the cholesterine from the blood, though its excretion is not entirely suspended. After death, but a small quantity of bile is found in the gall-bladder.

---

☞ Business letters to the Journal should be addressed to Dr. E. INGALS, Chicago, Ills., P. O. Drawer 5787. When money is received a receipt will be returned with the next number of the Journal, and subscribers failing to get such receipt will confer a favor by giving us notice of the omission.

---

Vaccine Matter.—Fresh and reliable Vaccine Matter may be obtained by enclosing One Dollar and addressing

Chicago Medical Journal,
P. O. Drawer 5787, Chicago, Ill.

